# Transforming a Pandemic Tool Into a Public Health Asset: Modified Delphi Study

**DOI:** 10.2196/77763

**Published:** 2026-09-16

**Authors:** Mohsen Malekinejad, Cesar Arturo Mendez-Lizarraga, Nicole Skehan, Alyssa Bercasio, Rakhee Palekar, William B Lober

**Affiliations:** 1 Institute for Global Health Sciences, University of California, San Francisco, 550, 16th St, 3406, Floor 3, San Francisco, CA, 94143, United States, 1 4154765494; 2 MITRE Corporation, McLean, VA, United States; 3 School of Nursing, University of Washington, Seattle, WA, United States

**Keywords:** exposure notification, preparedness, response, Delphi, method, pandemic, outbreak

## Abstract

**Background:**

During the COVID-19 pandemic, over 100 countries successfully deployed smartphone-based exposure notification (EN) apps alongside conventional contact tracing to mitigate the transmission of SARS-CoV-2. These systems enabled millions of COVID-19–positive individuals to anonymously alert community members of a potential exposure. Although evidence on EN program implementation is limited, leveraging expertise from public health professionals, academics, and global implementers from various disciplines can contribute to addressing this research gap.

**Objective:**

This study aimed to explore barriers and facilitators to adoption, identify optimal use cases for these tools, and understand how advancements in technology and policy might maximize EN utility for mitigating other infectious outbreaks.

**Methods:**

We used the Delphi method to develop a comprehensive online survey comprising closed-ended and open-ended questions. We considered consensus was reached when at least 75% of respondents selected “agree” or “strongly agree” for an item or statement. More than 30 global EN experts known to the investigators were invited to participate in the survey, and those who participated were asked to nominate or recruit others deemed eligible via a peer-to-peer method. We used descriptive and content analyses to summarize key findings from the survey. We invited the same experts to attend virtual focus groups to further probe their insights into the survey findings.

**Results:**

Twenty-two experts from 6 countries, including 10 experts from the United States, representing 3 continents, participated. Respondents were involved in the implementation of various EN technologies that encompassed varying levels of integration with existing public health agencies’ infrastructure. Consensus was reached concerning the development of additional functionalities of the system, such as the ability for users to self-report a positive result from a SARS-CoV-2 at-home test, receive additional public health information from the app (ie, information on testing and social services), and receive general venue-based notifications concerning superspreader events. Other items that reached consensus included how the tool could be used for additional epidemiological purposes, such as expanding the technology to other airborne diseases, and the need for integration with surveillance data streams to augment analytics and forecasting.

**Conclusions:**

Global EN systems have the potential to be optimized for public health emergencies beyond COVID-19 and can be used as epidemiological surveillance tools in future disease outbreaks. Decision-makers and experts should revise existing protocols and seek to incorporate new functionality to alleviate the disease burden on both users and public health agencies.

## Introduction

### Background

The COVID-19 pandemic placed unprecedented demands on the public health workforce. In response, digital contact tracing (DCT) and exposure notification (EN) tools were developed to enhance the efficiency of public health outbreak control, particularly during large case surges [1,2]. While some DCT systems were based on direct reporting, a new generation of more automated, scalable EN systems leveraged smart devices to alert individuals of a potential exposure to the SARS-CoV-2 virus, even without direct contact or relationship with the case. Implementation and evaluation of these EN systems within different jurisdictions were shaped by diverse system architectures, deployment strategies, and communication protocols, each influenced by political, social, and financial contexts. These tools also varied in their approach to privacy; some collected personal identifiers while others were completely anonymous and privacy-preserving [3].

### Proximity Detection Technologies

EN systems work by measuring distances between individuals, using various technologies, such as GPS and Bluetooth Low Energy, to determine if a user was in close contact of a person with a confirmed COVID-19 diagnosis [4]. GPS was used in multiple systems—including those in South Korea and China—because it can capture specific user information [5,6]. However, standard GPS is considered unreliable in determining the exact position of a person and requires a significant amount of battery power.

**Figure 1.**
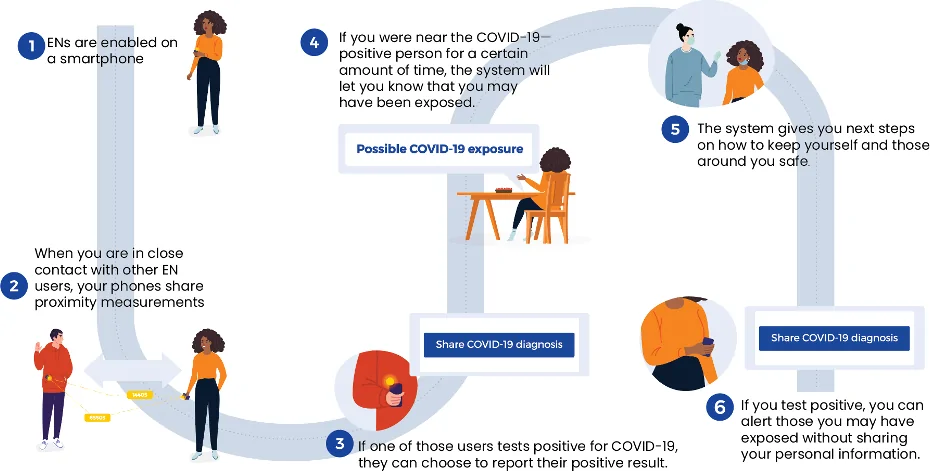
Overview of mobile exposure notification (EN) apps using the Google and Apple Exposure Notification (GAEN) System. Adapted from the California Department of Public Health and Aronoff-Spencer et al [13].

On the other hand, Bluetooth-based systems could not record an individual’s specific location and therefore were unable to identify where a person was exposed; however, they could assess the proximity between 2 individuals and provide greater privacy protection [7,8]. These systems relied on the generation of random digital codes; when 2 devices came close together, the codes were exchanged. By relying on 2 servers, a key server that stored keys from devices whose users reported positive tests and a verification server that checked for genuine positive tests, devices routinely searched for matches among stored codes. If a match occurred, the app alerted the user about a potential exposure, without disclosing who, when, and where the exposure took place [9,10]. Examples of Bluetooth-based EN systems include Singapore’s BlueTrace protocol—which was used in Singapore’s TraceTogether app and Australia’s COVIDSafe app—and Google and Apple’s jointly developed EN protocol, Google and Apple Exposure Notification (GAEN) system, which became the most widely adopted EN system during the pandemic. GAEN apps used Bluetooth Low-Energy signal exchanges between EN-enabled smartphones to estimate exposure risk based on duration and proximity to an individual’s smartphone who voluntarily disclosed a positive COVID-19 test result (Figure 1) [3,7,11,12].

Other proximity detection technologies used Wi-Fi or ultra-wideband systems, while others deployed a QR-based system or a combination of multiple approaches [8]. Some apps also used a combination of different technologies, such as India’s “Aarogya Setu” app, which used both GPS and Bluetooth [14].

### Centralized vs Decentralized Data Systems

Global EN systems also differed in whether they used centralized or decentralized protocols. Centralized protocols stored user interactions on a central server, which allowed for more meticulous epidemiologic tracking and analysis. These protocols enabled public health agencies (PHAs) to track second-order contacts and super-spreader events. Singapore’s BlueTrace system used a centralized system, as did China’s and South Korea’s apps [6]. However, centralized protocols were often considered controversial, given that they potentially enabled governments to access personal health data and therefore raised many user privacy concerns. Conversely, decentralized protocols determine and save proximity data and exposure risk entirely on a user’s mobile phone to preserve user privacy. While decentralized protocols made epidemiologic analysis difficult, they allowed for greater privacy of user data [7]. Apps created on the GAEN system prioritized user privacy over disease surveillance, using a decentralized protocol, while allowing PHAs to set risk parameters and public health messaging by jurisdiction, at the national or subnational level [13,15].

### The Future of EN

Most EN systems relied on government-sponsored servers to enhance cross-jurisdictional interoperability while preserving user privacy. However, PHAs varied in how they assessed exposure thresholds, further complicating comparisons across implementations [16,17]. While GAEN’s privacy-preserving model limited targeted public health messaging and robust impact assessments, modeling studies in settings with meaningful adoption rates—such as the United Kingdom and the states of Washington and California in the United States—suggest that digital EN systems averted significant numbers of new cases [11,12,16-28]. By contrast, non-GAEN systems, which collected identifiable data and location information, offered potential advantages in precision and public health follow-up. These systems could provide more tailored guidance to users and enable PHAs to assess the broader impact of exposures on public health. However, these systems often raised privacy concerns and faced barriers to adoption [2].

As the COVID-19 public health emergency concluded, scientists, policymakers, and public health leaders began to discuss both future and alternative uses of EN systems. Proponents highlighted the unprecedented collaboration between major technology companies and government institutions, resulting in the global deployment of EN systems, with over 300 million users worldwide [20,29-31]. Critics noted that while some countries and states deployed EN systems successfully, many regions either did not adopt the technology or failed to achieve meaningful user uptake. Despite variations in adoption and evaluation, EN systems demonstrated significant potential to augment infectious disease response and a remarkable ability to scale to large numbers of users and link jurisdictions, all while preserving privacy. As health systems prepare for future outbreaks, it is crucial to address gaps in EN design, deployment, and adoption to maximize the impact of these tools. It is also critical to understand the barriers and facilitators to their use to guide the development of policies and technologies that strengthen national and global health capacities.

We developed a modified Delphi study and engaged experts to explore barriers and facilitators to adoption, identify optimal use cases for these tools, and understand how advancements in technology and policy might maximize EN utility for mitigating other infectious disease outbreaks. By learning from the successes and limitations of EN systems during the COVID-19 pandemic, we seek to inform the design and implementation of more effective outbreak response tools for future use.

## Methods

### Study Design Overview

We used a modified Delphi Type 3 approach to gather expert knowledge on EN systems, using a comprehensive survey designed to capture both quantitative and qualitative data [32]. Appendix A in Multimedia Appendix 1 summarizes the study characteristics [33,34].

### Curation of Ideas to Inform Survey Development

We curated initial ideas based on the authors’ direct experiences implementing an EN system in California, as well as insights gathered from meetings of the Digital Notification Alliance (DNA), a US-based community of EN practitioners active during the COVID-19 pandemic, with participation from representatives of 16 states. To further develop our framework, we conducted a targeted literature search in PubMed and Google Scholar for relevant peer-reviewed articles and white papers during March-April 2023. The search focused on studies describing the implementation, evaluation, or adoption of EN systems and, when contextually relevant, other DCT tools during the COVID-19 pandemic. We used combinations of keywords such as “exposure notification,” “digital contact tracing,” “implementation,” “barriers,” “facilitators,” “COVID-19,” and “evaluation.” We searched for papers published between 2020 and 2023 in English. Publications were included if they discussed real-world implementation experiences, adoption barriers, or lessons learned from EN systems. The references identified through this process—together with relevant white papers and reports from our professional networks—were used to structure survey items and domains [3,5,9-11,17,20-22,24,26,28,29,35]. Additionally, we contacted our network of EN implementers across the United States and internationally to inquire about relevant publications and gray literature.

### Survey Development

Based on curated inputs, we developed a Delphi survey to collect expert insights on best practices, implementation challenges, and future directions for EN systems, with a focus on those using the GAEN protocol during the COVID-19 pandemic. The survey comprised six major sections: (1) participant consent and survey introduction; (2) expert demographics and professional background; (3) perceived successes, barriers, and facilitators of EN deployment; (4) perspectives on key system functionalities, disease applicability, macro-level governance, and considerations around privacy, equity, and communication; (5) opinions on potential uses of EN technologies beyond infectious disease outbreaks; and (6) participant demographics and peer referrals.

The final survey included approximately 100 questions, incorporating multiple-choice (including Likert-scale and rank-order items) and open-ended formats. Likert-scale items assessed the perceived importance or level of agreement on various implementation dimensions, while open-ended items allowed for elaboration and identification of additional considerations. The survey was administered via Qualtrics [36]. The instrument is available in Appendix B in Multimedia Appendix 1.

### Participant Recruitment

Using a combination of convenience and peer-referral methods, we recruited participants from our extensive network of US-based and international EN experts with whom we had collaborated and exchanged scientific ideas throughout the pandemic. We initially identified participants through the DNA network group to share lessons learned and best practices in EN implementation. Through the DNA network, we distributed invitations via its membership listserv, solicited referrals (snowball sampling), and leveraged its endorsement to broaden participation beyond our immediate collaborators. We also invited key authors of published papers on EN prior to the inception of our study. In total, 56 eligible experts representing PHAs, academia, and the technology industry were invited to participate. Eligibility criteria included being at least 18 years of age and having experience in the development, implementation, or evaluation of EN systems during the COVID-19 pandemic. Of the 56 identified experts, a total of 30 initially expressed interest in participating.

### Pilot Testing and Data Collection

Using QualtricsXM, we conducted a pilot test with 4 individuals to evaluate the survey’s logistics, length, content, and flow [36]. Based on feedback, we made minor revisions and then distributed the survey via email to 30 experts who had previously expressed interest in participating. The survey was launched in fall 2024, with up to 3 reminder emails sent at 7- to 10-day intervals. All responses were anonymous and untraceable after the pilot phase. Data from the pilot were included in the final data analysis.

### Data Analysis

We conducted descriptive analysis of the quantitative data using Microsoft Excel. For Likert-scale questions, we adopted the 75% agreement threshold that was suggested in the systematic review of reporting Delphi studies to establish a working definition of “large majority” for our study [34]. This threshold was used to identify items that were deemed more important or to represent a higher level of agreement among experts, but it should not be seen as a consensus threshold. For items or statements that required ranking, we used a weighted score to determine the order based on the number of responses for each question:


$$\begin{array}{rl} \text{Weighted score} & =[(\text{number of first place votes})\times 1.0]+[(\text{number of second place votes})\times 0.5]\\ \;+[(\text{number of third place votes})\times 0.33]+[(\text{number of fourth place votes})\times 0.25]\\ \;+[(\text{number of fifth place votes})\times 0.2]+[(\text{number of sixth place votes})\times 0.16]\\ \;+[(\text{number of seventh place votes})\times 0.14]+[(\text{number of eighth place votes})\times 0.12]\\ \;+[(\text{number of ninth place votes})\times 0.11] \end{array}$$

For the qualitative data, 2 researchers conducted a thematic analysis to identify recurring themes, which were used to supplement our analysis of the quantitative data. This allowed us to further elaborate on expert experiences and opinions, from which we could infer major key points and generate actionable recommendations. Examples are shown in Table 1.

**Table 1.** Thematic analysis of open-ended question responses^a^.

Themes and questions	Responses	Subtheme
Lessons from the COVID-19 pandemic		
Do you think your EN^b^ program achieved goals and objectives?	“It did so by complementing conventional contact tracing; compared to the latter, it led to an estimated 5%‐10% increase in the number of cases detected.”	Public health
The next EN application		
Would you consider continuing EN programs if COVID-19 resources were available?	“EN systems should ideally run continuously, even when there is no immediate need. That way, it can be ready to scale up quickly when needed.”	Public health
Do you have suggestions as to what governance principles and procedures should be in place prior to the next implementation of EN systems?	“International coordination between public health agencies and tech companies is necessary to iron out interests and articulate norms that can guide decisions.”	Public policy
Are there ways to improve the opt-in sharing of information?	“There should be levels of information sharing for the comfortability of opting into the system..., but there should be no requirement for it.”	Technical features

a The themes included in the survey are shown in the first column. Based on question types and responses, 3 subthemes emerged: technical features of EN applications, public health, and public policy issues.

b EN: exposure notification.

### Expert Review and Validation of Findings and Final Recommendations

Following the initial round of data analysis, all expert participants were invited to a live, 2-hour virtual presentation to review findings and provide additional insights. Two sessions were conducted, and discussions were recorded with participants’ permission. One researcher took detailed notes from the sessions and analyzed participant feedback. Comments and suggestions from these sessions were integrated into the final interpretation of results. The final recommendations were developed through a multistep process. Topics and ideas that were deemed important or reached a high level of agreement in the quantitative survey were prioritized. These were further enriched by qualitative feedback provided by participants and subsequently validated through the live expert reviews and discussions.

### Ethical Considerations

Our study protocol was reviewed and approved by the Committee on Human Research at the University of California, San Francisco (UCSF), through an expedited review process (IRB number: 23-39886; reference number: 398640). The study did not include personal or sensitive questions and focused instead on gathering expert opinions about a digital health product. All data were stored securely within UCSF’s protected data systems and reported only in aggregate form to safeguard participant privacy. Participants could choose to share their general geographic location for national-level analysis. Those who provided informed consent were invited to complete the survey and received a US $50 gift card for participating in at least 1 round of data collection. Individuals who also took part in the live discussion received an additional US $100 gift card.

## Results

### Demographics

A total of 22 individuals from 3 World Health Organization (WHO) Regions (Americas, Europe, and Western Pacific), representing 6 countries, participated in our Delphi. We asked participants to select their involvement with EN systems during the pandemic, as all had carried out more than one specific activity (Table 2). The most common roles included data analysis (n=19, 86.3%), coordination with public and private partners (n=17, 77.2%), and elaborating policies and guidelines for decision-makers (n=14, 63.6%; Table 3).

**Table 2.** Participants’ primary country for exposure notification (EN) implementation, years of experience with EN, and other characteristics of EN implementation (N=22).

Characteristics	Values, n (%)
Primary country in which participants were involved in EN^a^ implementation	
United States of America	10 (45.5)
Belgium	2 (9.1)
United Kingdom	2 (9.1)
Germany	1 (4.5)
Singapore	1 (4.5)
Italy	1 (4.5)
Unknown	5 (22.7)
Years of experience in EN systems	
1‐3 y	11 (50.0)
3 or more years	10 (45.4)
Not reported	1 (4.5)
Type of EN system first implemented	
Customized GAEN^b^ app	8 (36.3)
ENX (Exposure Notification Express)	5 (22.7)
Fully custom app	5 (22.7)
Mixed or hybrid model	1 (4.5)
Other	2 (9.1)
Unknown	1 (4.5)
Integration of EN systems to PHA^c^ public health response	
Not entirely integrated	9 (40.9)
Fully integrated	7 (31.8)
Stand-alone systems	2 (9.1)
Other	1 (4.5)
Unknown	3 (13.6)
Main implementer of EN systems in jurisdiction	
Mixed model	11 (55.0)
Health departments	7 (35.0)
Contract with private entity	2 (10.0)
Not reported	2 (9.1)

a EN: exposure notification.

b GAEN: Google and Apple Exposure Notification system.

c PHA: public health agencies.

**Table 3.** Participants’ areas of expertise and roles adopted in the exposure notification (EN) implementation^a^.

Areas of expertise and roles adopted	Values (n)
Areas of expertise	
Public health	15
Epidemiology	14
EN^b^ systems and technology	12
Policy	5
Health communications	4
Engineering	4
Research	10
Other	8
Patient care	2
Responder’s role in EN implementation	
Data analysis	19
Coordination with public and private partners	17
Health communication media	13
Program implementation	12
User experience	11
Interface design	8
Systems development	12
Policies and guidelines elaboration for decision-makers	14

a Respondents (N=22) were asked to select all options that applied to their profile.

b EN: exposure notification.

### Lessons From EN System Implementation During the COVID-19 Pandemic

#### Overview

**Figure 2.**
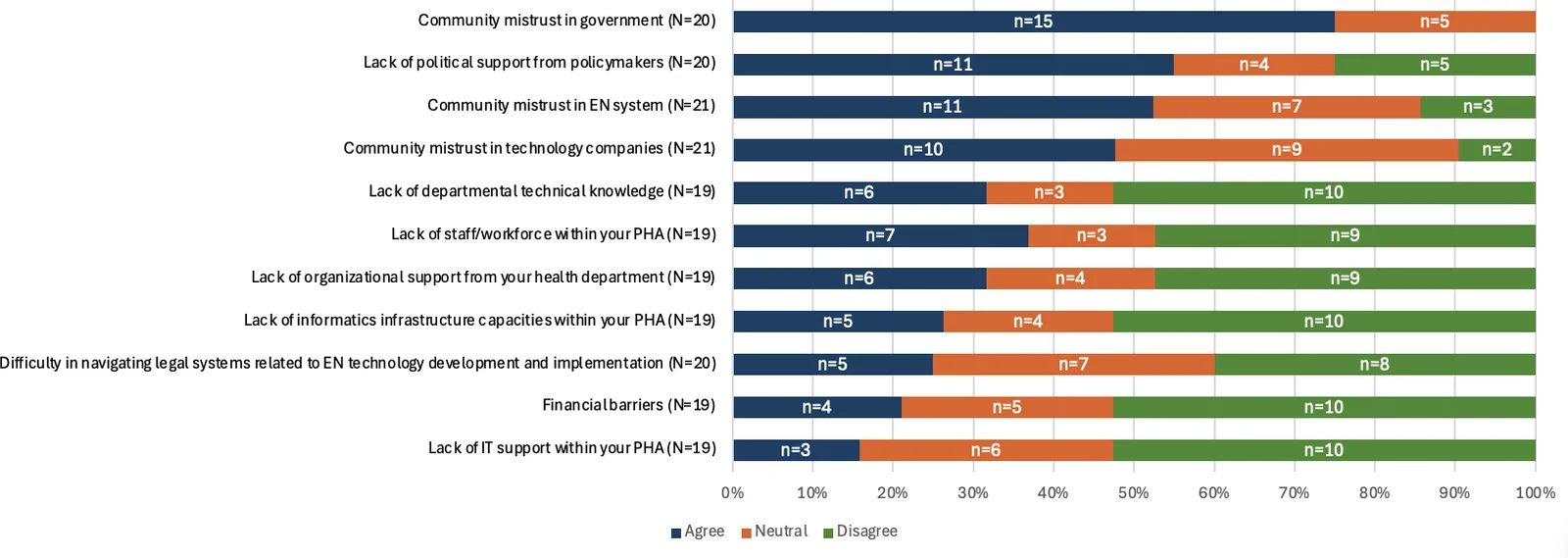
Barriers to exposure notification (EN) program implementation during COVID-19. Response statements have been shortened for practical reasons. Agreement and disagreement percentages are aggregates of “agree,” “strongly agree,” “disagree,” and “strongly disagree,” respectively. Statements are ranked from the highest to the lowest percentage of agreement.

When inquired about specific barriers to EN program implementation, the large majority of participants (15/20, 75% of respondents) identified “community mistrust in the government” as a major barrier (Figure 2). Items voted by at least half of the participants as barriers to implementation included the following: the lack of political support from policymakers (11/20, 55%) and community mistrust in EN systems (11/21, 52.4%). Other items voted by less than 50% of respondents as barriers included, but were not limited to, the following: community mistrust in technology companies (10/21, 47.6%), lack of staff workforce within PHAs *(*7/19, 36.8%*)*, and lack of departmental technical knowledge (6/19, 31.6%).

#### Implementation Barriers

Respondents pointed out a combination of factors such as “lack of awareness, privacy concerns, government mistrust, and lack of understanding of how the app worked” as barriers to PHA implementation. For others, a bigger budget could have translated into “continuous development, better privacy features, and relevant data analysis.” In the case of the United States, the lack of a centralized system caused a “significant burden on jurisdictions.” In addition, we inquired about ideal resources (framed as a “Wishlist”) for better programmatic and health outcomes. Respondents indicated a desire for higher budgets for increasing user engagement, stronger analytical resources for tracking relevant metrics, access to testing without symptoms, full support from decision-makers, an opt-out modality, central collection of outcome data (eg, test results for notified contacts), better integration between testing and treatment resources (as well as with other agencies), more app promotion, and a “multilateral dialogue on EN system design principles with tech giants with a deference to public health.”

#### Program Goals

When inquired whether participants thought their EN programs achieved proposed goals and objectives, some stressed that they did so by “alerting hundreds of thousands of people of exposures,” while others expressed that the system notified “far more contacts per exposure than traditional contact tracing.” Conversely, some believed they did not achieve their goals and objectives because they faced little adoption, late integration into their health authorities’ processes, and “lack of public communication about the processes of the app.”

### Epidemiological and Clinical Considerations for Future EN Systems

With respect to the applicability of proximity-based EN apps for infectious diseases, the large majority of respondents labeled airborne diseases as “very applicable” (17/21, 80.9%), while fomite-transmitted diseases were considered “very applicable” by less than half (9/20, 45%). On the other hand, blood-borne diseases and sexually transmitted infections were predominantly considered as “not very applicable” by 80% (16/20) and 65% (13/20) of respondents, respectively. Concerning the incorporation of diseases into future EN apps, the large majority of participants considered COVID-19 (20/22, 90.9%) and measles (17/22, 77.3%). Other diseases that were considered by at least half of the respondents were influenza (15/22, 68.2%) and tuberculosis (13/22, 59.1%). Additional pathogens that were suggested in the written comments by participants included respiratory syncytial virus, meningococcal meningitis, Disease X, HIV, hepatitis A, Mpox, and other sexually transmitted infections.

Finally, we asked participants to rank key epidemiological and clinical characteristics of pathogens that should be considered for future app developments, which resulted in the following order of priorities: (1) transmissibility of the pathogen (incubation period, infectiousness, etc; 82 points); (2) available protective actions (75 points); (3) disease burden (71 points); (4) level of community immunity (67 points); and (5) economic availability (or feasibility) to take protective actions (65 points).

### Public Health and Implementation Considerations for Future EN Systems

#### EN Value for the Future

When asked if there was any value in continuing EN programs after the end of the COVID-19 pandemic as a public emergency of international concern, respondents agreed that there is a need to be ready: “waiting for an emergency will always be too late...even modest benefit with ongoing use is worthwhile.” Pandemic preparedness and response require a set of continuous actions, and for EN systems, “ideally they should run continuously...ready to scale up quickly when needed.” A challenge highlighted was the need to demonstrate its value to secure the necessary resources and political support, with one participant noting, “we need to demonstrate value to enable resource allocation and political support.”

#### Strategies to Improve EN Adoption

Clear communication—such as the benefits for the individual and community, goals, and privacy issues—is key, as is open communication about any uncertainties (ie, “letting the wider public know what we don’t know”). On communicating with users about their exposure, messages need to be accurate and readable. As shared by the group, “information has to be credible and accessible.” Another suggested strategy was to include venue-based exposure alerts. On how to best leverage experience from implementors, a shared answer was to create standardized guides for PHA EN deployment, covering design, development, implementation, evaluation, and other aspects encompassing a public health intervention.

#### Communication Campaign

When participants were asked to select topics that should be considered for public-facing communication campaigns, topics deemed “very important” by the large majority were as follows: reminders to use the system when testing positive (18/22, 81.8%) and that users should directly benefit from using the system (17/22, 77.2%). Items that were selected by more than 50% but were below the large majority threshold included how to use the system (15/22, 68.1%), community benefits from using the system (13/22, 59.1%), and user privacy protection (13/22, 59.1%). Marketing strategies for EN technologies through public agencies included using social media and communicating to the broader public while advertising the app as “a tool for citizen science” to strengthen societal preparedness. About reducing technological disparities, “using free standalone devices” that can provide text-based notifications and inform of actions that communities can take was identified as the most common approach. Finally, when inquired about the utility and importance of a comprehensive and multifunctional EN app for future public health threats, participants stated that “it may lose focus on what the main priority...and users may become overwhelmed,” while a participant suggested that EN apps should be limited to infectious disease monitoring and control.

### Technical Features Considerations for Future EN Systems

#### General Technical Features for Future App Development

**Figure 3.**
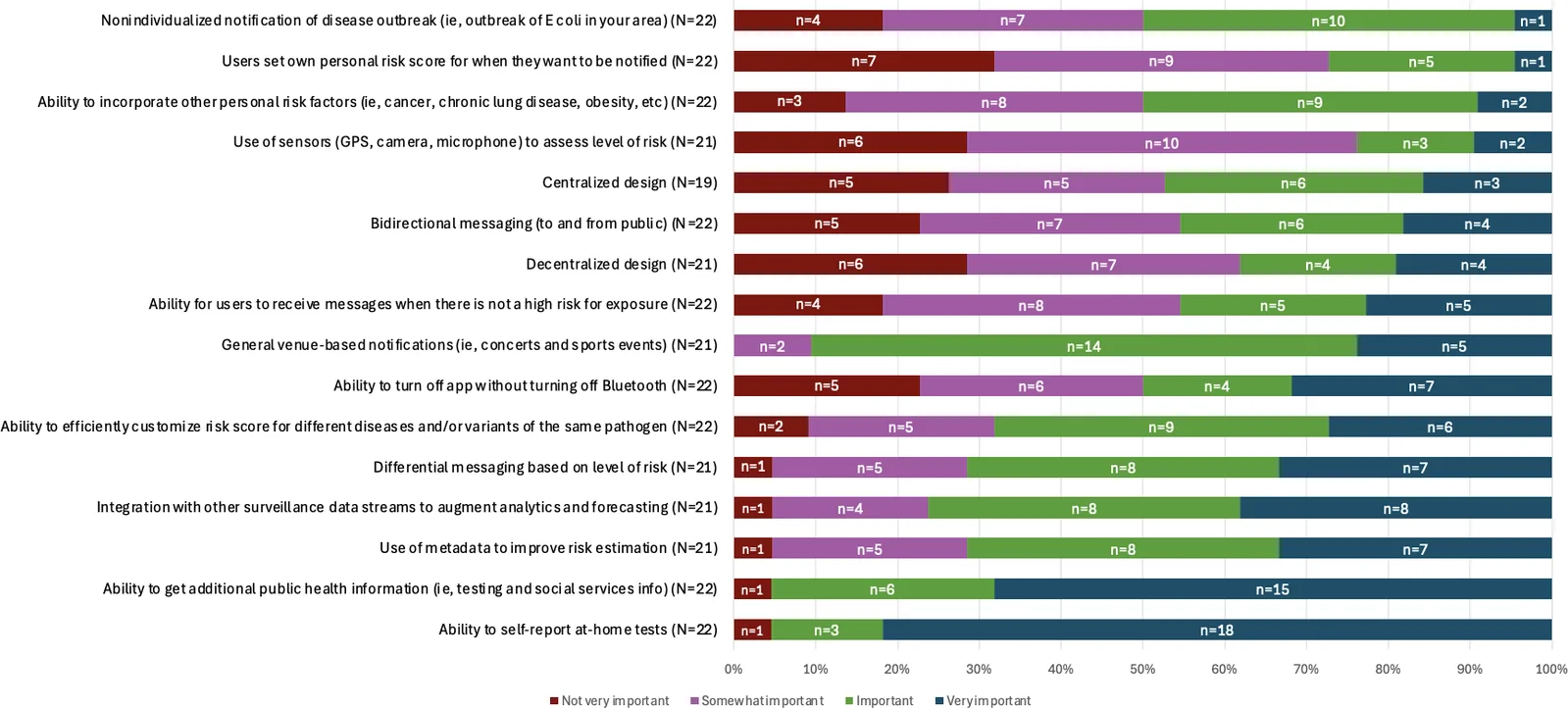
System functionalities and characteristics for the next exposure notification (EN) app are shown together by their level of importance on a 4-point Likert scale. Items are ranked based on the percentages obtained for “very important” from respondents (n=22).

Participants were asked to label the importance of functionalities and characteristics for future EN systems. The ability to self-report at-home tests was the only item that was considered “very important” by the large majority (18/22, 81.8%) of respondents, while the ability to get additional public health information was considered “very important” by 68.2% (15/22). Also, the venue-based notifications feature was labeled as “important” by more than half (14/21, 66.7%) of participants. Features like the use of sensors to assess the level of risk and the ability to set a personal risk score for notifications were categorized as “somewhat important” by almost half of the participants. No feature was deemed “not very important” by one-third or more of respondents. The level of importance of all functionalities is shown below (Figure 3).

#### Specific Technology for Proximity Detection

Half of the participants considered mixed EN signaling technologies to be the best solution for future developments, followed by only Bluetooth Low Energy (5/22, 22.7%) and ultra-wideband (2/22, 9.1%). Mixed EN technology examples included combining Bluetooth Low Energy with other methods (ie, GPS or venue-based alerts), and participants commonly wrote down “Bluetooth plus Ultra-wideband system” as another option. Nearly 20% (4/22) of respondents did not offer a specific opinion.

When participants were asked to rank strategies to ease user burden, the order is as follows: (1) easier methods for activation (97 points), (2) increased access to other public health resources (86 points), (3) more accessible language (85 points), (4) different user workflows (73 points), (5) new methods for direct user communication (50 points), and (6) new EN hardware (29 points). Furthermore, the group was divided on whether the use of custom hardware or devices should (10/21, 47.7%) or should not (11/21, 52.3%) be incorporated. Further insight on this revealed that it has the potential “to be quickly deployed in outbreaks in populations without smartphones” but made no sense in settings where mobile phone coverage is high, as “it seems unnecessary to ask them to carry another device.”

#### Privacy

Out of 4 options (accuracy of EN, perceived risk by users and their willingness to adopt, scalability tradeoff, and system evaluation capabilities), we asked participants to identify the option that is more important to consider for future EN development in relation to the level of privacy. Privacy was the only characteristic deemed as “more important” by the large majority against the perceived risk by users and their willingness to adopt (18/22, 81.8%). Privacy was voted as “less important” by more than half of participants and less than the large majority for the following: accuracy of EN (13/22, 59.1%) and system evaluation capabilities (12/21, 57.1%). Finally, privacy was neither more nor less important for the scalability tradeoff (50% vs 50%; N=20). The use of technology with a Bluetooth-based protocol that does not require information about the location of the user was considered advantageous, as “it preserved privacy while not creating a huge burden on user batteries—unlike GPS—...with the downside of not fully understanding the location of cases.” Concerning how users should share information, participants felt that “there should not be a requirement for data sharing” and that users should be provided with layers or levels of data sharing that users can choose to opt into, allowing users to exercise autonomy.

### Institutional and Governance Considerations for the Future EN Systems

Concerning where to place EN programs in organizational structures, more than half opted for a hybrid model (eg, sharing responsibilities between emergency response and data informatics; 13/22, 59.1%), leaving out emergency response (5/22, 22.7%), data systems-information (2/22, 9.1%), and contact tracing programs (2/22, 9.1%) as potential sites. Other proposed locations included disease surveillance and biosecurity within the military structure. Another response suggested a more restrained approach: “discussing the benefits and challenges with agencies, as it requires an interdisciplinary approach.”

On governance for EN programs, some acknowledge that it should be on a country level and that “a regulatory framework should be in place before the next public health emergency.” A considerable proportion of participants (13/21, 61.9%) expressed that implementation levels should be nationwide, with an option for states to customize.

International and national institutions that should be reached out to for future collaborations include the WHO (14/34, 41.2%), National Institutes of Health (9/34, 26.4%), and Federal Emergency Management Agency (8/34, 23.5%), as well as other agencies such as Centers for Disease Control and Prevention, Homeland Security, Food and Drug Administration, European Center for Disease Prevention and Control, and central hubs from around the world. When asked to rank institutions that should be tasked with building trust, the following order emerged: (1) public health departments (79 points), (2) EN system-technologies (66 points), (3) state government (64 points), (4) technology companies (54 points), and (5) federal governments (52 points). With respect to organizing working groups (such as DNA in the United States), respondents expressed that these groups should continue to meet and plan for the next steps, as “it fosters relationships with private and public partners.*”* The most effective way to use these groups is to leverage their expertise in designing next-generation tools.

The importance of working with smartphone manufacturers was emphasized by the large majority of the group (17/22, 77.2%), as some pointed out that in addition to tapping into their technical expertise, “it helps build relationships and improve operations that benefit actors.” When examining the possibility of working with other companies aside from Google and Apple, participants expressed that mobile phone and app companies should be included, though “many settings are regulated by these two companies...both need to open up their ecosystem and infrastructure that allows others to work*,*” highlighting the support from big tech companies.

## Discussion

### Summary of Key Findings

To our knowledge, this is the first study to systematically capture insights from experts on the future of EN systems. Although others convened group discussions for similar purposes, those attempts were not as comprehensive as ours, were not peer-reviewed, and were convened or led by industry personnel [35]. Our study suggests that during the COVID-19 pandemic, the main barriers to EN program implementation included community mistrust in technology companies and mistrust in EN systems, followed by a lack of political support from policymakers. Airborne diseases, such as measles, influenza, respiratory syncytial virus, and tuberculosis, were considered the most appropriate group of diseases to incorporate into EN systems. Additional features to consider for the next app include pathogen transmissibility, protective measures, and the burden of disease in the community. Some of the most important features and system characteristics for future apps were the capacity to self-report at-home tests, access to additional public health information, general venue notifications, and differential messaging based on risk level. Finally, for public policy purposes, the study highlights the need to further discuss the benefits and challenges with involved agencies, as it requires collaboration within and across institutions, and efforts should be made to foster working groups to enhance EN systems.

### Public Health and Policy Implications and Recommendations

This study serves multiple purposes. The first is to describe barriers experienced during the COVID-19 pandemic that require action for improving performance and facilitating the implementation of future EN systems. The second is to provide a landscape of improvements to be considered for the next batch of EN systems. These improvements build upon the experience participants shared and make a call for intersectoral collaboration across public health professionals, the technology industry, and policymakers with key roles in advancing preparedness and response against infectious diseases across all government levels, civil society, and academia. Third, it advocates the preservation of current technologies to enhance public health functions by expanding its scope of work to other infectious diseases and by promoting working groups to leverage expertise (regardless of geographical dimensions), develop guidelines, and foster networks across sectors. Finally, it lays out recommendations for decision-makers, public health professionals, and the technology industry for enhancing the next generation of EN systems as follows:

First, efforts to strengthen EN systems should address key implementation and adoption barriers. Legal barriers should be identified and resolved first to facilitate timely technological development and deployment. At the same time, community trust should be built via close partnership with public health authorities at local, state, and national levels. Furthermore, close collaboration with technology companies is critical to develop strategic work plans aimed at strengthening user trust and increasing app adoption.

Second, to enhance the functionality and effectiveness of EN apps, participants recommended incorporating several core epidemiological and clinical features. These include information about the transmissibility and natural history of the pathogen, clear guidance on protective actions that individuals can take—both pharmacological and nonpharmacological—as well as information about available economic resources to support those actions. Including contextual information about disease burden was also considered important. In addition, experts supported expanding EN systems beyond COVID-19 to other high-burden pathogen groups, including airborne diseases, vaccine-preventable diseases, and sexually transmitted infections.

Third, several system-level and user-level characteristics were identified as priorities for future development. EN systems should enable general venue notifications and provide differential messaging based on individual risk level. The use of metadata to improve risk estimation and the integration of other surveillance data sources to enhance analytics and forecasting were also recommended. At the user level, functionality should allow individuals to self-report at-home test results, request additional public health information, incorporate individual-level risk factors, and receive notifications that clearly indicate exposure risk level (eg, high or low). Participants also emphasized that users should be able to turn off the app regardless of Bluetooth status. To improve access, EN systems should incorporate easier methods for app activation, expand access to other public health resources, and improve accessibility by offering multiple language options.

Finally, to support sustainable implementation, experts recommended fostering structured working groups that include local and national public health departments and other relevant organizations. These groups should create subcommittees focused on specific topics, use standardized approaches to estimate key performance indicators, organize workshops to equip implementation teams with practical tools, and systematically document and share lessons learned. Finally, planning, implementation, and evaluation of EN systems should involve coordinated engagement with public health departments, EN technology developers, state and local governments, technology companies, and, when appropriate, federal agencies.

### Comparison to Prior Work

Although no prior peer-reviewed study has examined the same breadth of EN implementation barriers, disease applicability, future system functionality, and governance priorities in one place, some studies addressed certain aspects of our findings. For instance, other studies also noted that adoption of EN and DCT apps was influenced by privacy concerns, trust, perceived usefulness, and usability [6,37]. Furthermore, another paper emphasized that EN deployment depended on close collaboration among technology companies, developers, and public health authorities, while fragmented rollout and limited data collection complicated effectiveness assessment [7]. Our findings also reinforce the conclusions from the best-practice guidance and implementation experiences from several European countries: EN apps should rapidly connect users to testing, quarantine guidance, and other public health resources, and risk-stratified messaging and actionable recommendations may improve both engagement and public health utility [8]. Implementation experiences in Finland also suggested that EN systems function best when integrated into broader public health response activities rather than operating as stand-alone notification tools [38]. Our findings on future system characteristics suggest integration with surveillance data—extending prior work that proposed standardized evaluation metrics for EN systems [13] and suggested that EN activity may support short-term outbreak forecasting and surveillance functions [39]. Finally, our recommendation for structured working groups and stronger cross-sector coordination is reinforced by lessons from collaborative work in the United States during the COVID-19 pandemic, such as the MIT Lincoln Laboratory initiatives [35] and DNA [40], which emphasized iterative assessment, governance mechanisms, and sustained collaboration among PHAs, policymakers, and technology partners.

### Caveats and Limitations

Although this study is the first to systematically gather and synthesize data to outline actionable steps for strengthening EN systems, the findings should be interpreted with caution. First, our sampling strategy and modest sample size limit generalizability, particularly because most participants were based in the United States and only about half reported direct implementation experience.

Second, this study employed a type 3 Delphi approach to gather expert perspectives on enhancing EN systems as part of infectious disease response. By design, our modified Delphi approach included only a single round, which constrained our ability to capture a more iterative or comprehensive assessment. Unlike a type 4 Delphi that seeks structured consensus over multiple rounds, our goal was to surface areas of opportunity and identify priorities as health systems prepare for emerging and reemerging infectious disease outbreaks. Additional work is needed to further refine these priorities and inform next steps for advancing EN technologies.

Finally, although our analysis was not explicitly structured around an implementation science framework, the barriers and facilitators identified map closely onto commonly used domains—such as contextual readiness, stakeholder engagement, and system integration—and can guide future research that applies models like the CFIR (Consolidated Framework for Implementation Research) or RE-AIM (Reach, Effectiveness, Adoption, Implementation, and Maintenance) to digital public health interventions.

### Conclusion

EN systems have the potential to play a broader role in supporting ongoing public health responses to outbreaks beyond COVID-19. Our findings suggest that there are opportunities to adapt EN technologies into more flexible tools for future outbreaks. Achieving this mandate will require revisiting existing protocols, improving core functionalities, and addressing practical barriers that limit usability and integration with routine public health activities and cross-sectoral coordination.

Despite its limitations, our study helped identify priorities as a starting point for a roadmap to strengthen EN systems as part of preparedness for emerging and re-emerging infectious diseases. With system improvements, stronger coordination between technology and public health leaders, and continued evaluation, EN technologies can evolve into a more reliable and sustainable component of modern outbreak response.

## Supplementary material

10.2196/77763 Multimedia Appendix 1 Supplementary study quality characteristics and Delphi survey questions.

## ABBREVIATIONS

DCT: digital contact tracing
DNA: Digital Notification Alliance
EN: exposure notification
GAEN: Google and Apple Exposure Notification system
PHAs: public health agencies
UCSF: University of California, San Francisco
WHO: World Health Organization
