# Supplementary material for: Transforming a Pandemic Tool Into a Public Health Asset: Modified Delphi Study
Source: JMIR Public Health Surveill. 2026 Sep 16;12:e77763. doi: 10.2196/77763 (PMC13581147; doi:10.2196/77763)
Supplement: Supplementary file 1 [file publichealth-v12-e77763-s001.docx]

**APPENDIX**

A. **Study quality characteristics**

| Quality characteristic | Our study |
| --- | --- |
| Literature review conducted | Yes |
| Background information provided to respondents | Yes |
| Purpose is item generation or ranking or both | Yes |
| Number of respondents indicated | Yes |
| Number of respondents for round 1 indicated | Yes |
| Number of respondents for round 2 indicated | NA |
| Number of respondents for round 3 indicated | NA |
| Criteria used for respondents reproducible* | Yes |
| Polling described | Yes |
| Private decisions collected (anonymity) | Yes |
| Formal feedback of group ratings | Yes |
| Number of rounds conducted 2 or more | No |
| Number of rounds to be performed stated* | No |
| Predetermined definition of consensus | NA |
| Consensus forced | NA |
| Were criteria used for dropping items clear* | No |
| Stopping criteria other than rounds specified* | No |

Quality characteristics of the study (ref numbers)

*Indicates criteria from Diamond et al

B. **Survey**

EN Survey

Start of Block: Section 1: Survey Overview and Consent

T1.1 **Section 1: Survey Overview and Consent**

T1.2 Thank you for participating in this survey.

 The information gathered from this questionnaire is part of an iterative process to identify areas of high priority for immediate and long-term public health action. The purpose of this stepwise Delphi survey is to inquire the input and opinions of subject matter experts (SMEs) on the following:

 • The implementation challenges and successes of exposure notification (EN) systems during the COVID-19 pandemic.
 • Future features of digital applications that can be used for pandemic response, policy, and political considerations.

 In this study, data will be collected through the Delphi Methodology using Qualtrics, an online survey tool. This survey is considered to be the first round of three. Data will be collected, and responses will be confidential. For this survey administration, participants are invited to respond to and suggest additional topics for each category. After responses are collected, the data will be analyzed for frequency of responses, the survey will be modified based on the results, and suggested topics will be added (Note: new topics will only be added to the second round of the survey). The results will be summarized and disseminated among participants for review. This process will repeat until the completion of the third round or if consensus is reached. If consensus is not reached by the third round, participants will be invited to a discussion forum with the goal of reaching consensus.

 Objective: The principal purpose for the collection of this information is to aggregate the experiences of EN experts involved in the implementation, communication, and policy decision making efforts concerning digital EN systems. This survey places particular emphasis on those with experience utilizing the Google Apple Exposure Notification (GAEN) system during the COVID-19 pandemic. The goal is to create consensus on the most affective designs and deployment methods necessary to optimize the public health benefits of such a system and to provide recommended improvements where appropriate. This information is intended to be shared with public health institutions to assist them in their development of successful EN systems capable of rapid deployment for use in future pandemic responses.

Q1.1 Participation in this survey is voluntary and takes approximately 60 minutes. Subjects may stop participating at any time or may skip survey questions if they are not applicable to the subjects’ experience. Response options will include both open-response and Likert-scale questions to determine EN priorities. Basic demographic data will also be collected, as well as information that can help to assess the level of a participant’s expertise (e.g., number of years in their respective professional, years of education, etc.). The information collected shall be confidential and shall be collected and maintained in a manner that protects your privacy.

If you consider your professional training and expertise applicable to the subject matter of this global consensus statement project, we encourage you to participate.

By clicking, the “I accept” button, you are confirming that you have read and agree with the information above and you consent.

- I accept (1)
- I do not accept (2)

Skip To: End of Survey If Participation in this survey is voluntary and takes approximately 60 minutes. Subjects may stop p... = I do not accept

Display This Question:

If Participation in this survey is voluntary and takes approximately 60 minutes. Subjects may stop p... = I accept

Q1.2 In addition to completing the survey, the study team may need to contact you to further discuss and expand upon the topics in the survey through a 30-45min virtual interview. All data we collect will be kept confidential, and we will not share your personal information with anyone outside the research team.

By clicking, the “I accept” button, you are confirming that you have read and agree with the information above, and you consent to being contacted for an interview if additional follow-up is needed.

- I accept (1)
- I do not accept (2)

End of Block: Section 1: Survey Overview and Consent

Start of Block: Section 2: Characteristics

T2.1 **Section 2: Characteristics of Exposure Notification (EN) Subject Matter Experts (SMEs) and Public Health Jurisdiction**

Q2.2 What is your formal job title? Please describe below_________

Q2.3 What is your area of expertise? (Select all that apply)

- Public health (1)
- Epidemiology (2)
- EN systems and technology (3)
- Policy (4)
- Health Communications (5)
- Engineering (6)
- Research (7)
- Other or if you would like to provide more detail to your area of expertise (please describe) (8)__

Q2.4 How many years of experience do you have in your area of expertise?

- Less than 1 year (1)
- 1-5 years (2)
- 5-10 years (3)
- 10-15 years (4)
- 15-20 years (5)
- 20 years or more (6)

Q2.5 How many years of experience do you have in EN systems?

- No experience (1)
- Less than 1 year (2)
- 1-2 years (3)
- 2-3 years (4)
- 3 years or more (5)

Q2.7 How many years of experience do you have in pandemic response?

- Less than 1 year	(1)
- 1-2 years	(2)
- 2-3 years	(3)
- 3 years or more (4)

Q2.8 What is your role in pandemic response? (select all that apply)

- Surveillance (1)
- Development of guidelines (27)
- Policy development (28)
- Case Investigation (29)
- Strengthening of health systems (30)
- Vulnerable populations/gender (31)
- Contact tracing (32)
- Direct patient care (33)
- Laboratory (34)
- Vaccine development (35)
- Distribution of vaccines and other supplies (36)
- Technology development (37)
- None (38)
- Other (please describe) (39)

Skip To: End of Survey If How many years of experience do you have in EN systems? = No experience

Q107 What was the role you played in EN systems? (select all that apply)

- Development of public health guidelines for EN deployment	(1)
- Development of guidelines for decisions regarding EN system configuration (4)
- Policy development	(5)
- Systems development (6)
- User interface design (7)
- User experience (8)
- Program Implementation	(9)
- Data & Analytics (10)
- Health communication/media/marketing (11)
- Coordination with public and private partners (12)
- Analysis and presentation of data and trends (13)
- None (14)
- Other (please describe) (15) __________________________________________________

Q2.9 If you worked in a public health authority (PHA), what was the population size of your jurisdiction?

- Less than 500 thousand residents (7)
- 500 thousand – 1 million residents (8)
- 1-5 million residents (9)
- 5-10 million residents (10)
- Greater than 10 million residents (11)

Q2.12 What type of EN system was first implemented in your jurisdiction?

- GAEN Customized	(1)
- ENX	(2)
- Fully custom app	(3)
- Mixed/Hybrid model	(4)
- Other (please describe) (5) __________________________________________________

Q2.13 If you selected “mixed/hybrid model”, please describe the model below.________________

Q2.14 Did you transition to another EN system?

- Yes	(1)
- No (2)

Display This Question:

If Did you transition to another EN system? = Yes

Q2.15 If you selected “yes,” please select the EN system you transitioned to

- GAEN Customized	(1)
- ENX	(2)
- Fully custom app	(3)
- Mixed/Hybrid model	(4)
- Other (please describe) (5) __________________________________________________

Q2.16 When was the EN system turned on? If your jurisdiction launched a pilot EN system, please write an approximate date of when the pilot system was turned on.

|  | Month | Day | Year |
| --- | --- | --- | --- |
|  |  |  |  |
| Please Select: (1) | ▼ January (1 ... December (12) | ▼ 1 (1 ... 31 (31) | ▼ 1900 (1 ... 2049 (150) |

Q2.17 What was the reason for using an EN system? (select all that apply)

- Supplement manual contact tracing and case investigation system	(1)
- Replace manual contract tracing and case investigation system	(2)
- Other (please describe) (3) __________________________________________________

Q2.18 Was your EN system disabled?

- Yes	(1)
- No (2)

Display This Question:

If Was your EN system disabled? = Yes

Q2.19 When was the EN system disabled? Please write an approximate date.

|  | Month | Day | Year |
| --- | --- | --- | --- |
|  |  |  |  |
| Please Select: (1) | ▼ January (1 ... December (12) | ▼ 1 (1 ... 31 (31) | ▼ 1900 (1 ... 2049 (150) |

Display This Question:

If Was your EN system disabled? = Yes

Q2.20 What was the reason for disabling the EN system? (select all that apply)

- Financial barriers (1)
- Shutdown of servers (i.e.; APHL servers in the US) (9)
- Lack of organizational support (2)
- Lack of political support	(3)
- Lack of informatics infrastructure capacities	(4)
- Lack of workforce capacity	(5)
- Was deemed not to provide any benefits given change of the epidemiological pattern	(6)
- Other; please describe (7) __________________________________________________

Q2.21 Would you consider continuing the EN program if COVID-19 resources were available? Would you see value in continuing if the resources were not a concern?

- Yes	(1)
- No (2)

Q2.22 Please explain your answer to the previous question.

Q2.23 What type of server was used?

- Association of Public Health Laboratories (APHL)	(1)
- Federated Gateway System (EFGS)	(2)
- Your PHA’s own local server	(3)
- Other (please describe) (4) __________________________________________________

Q2.24 How was your EN system integrated into their public health response?

- Standalone systems	(1)
- Not entirely integrated	(2)
- Fully integrated	(3)
- Other (please describe) (4) ________________________________________________

Q2.25 Who was the main implementer of the EN system?

- Health department	(1)
- Contract with private entity	(2)
- Mixed model (please describe) (3)_________________________________________________

Q2.26 How would you describe the level of community support in the following?

|  | Very Low (1) | Low (2) | Moderate (3) | High (4) | Very High (5) | N/A (6) |
| --- | --- | --- | --- | --- | --- | --- |
| Mass Vaccination (1) |  |  |  |  |  |  |
| Shelter in Place Orders (2) |  |  |  |  |  |  |
| Masking Orders (3) |  |  |  |  |  |  |
| Quarantine/Isolation guidance (4) |  |  |  |  |  |  |
| Testing guidance (5) |  |  |  |  |  |  |

End of Block: Section 2: Characteristics

Start of Block: Section 3: Identifying Successes, Barriers, and Facilitators of EN Systems

T3.1 **Section 3: Identifying Successes, Barriers, and Facilitators of EN Systems**

T3.2 **Identifying Successes**

Q3.1 What is the approximate percent cumulative adoption of your EN system? Please use the total population size of your jurisdiction as the denominator.

- Less than 20%	(1)
- 20-30%	(2)
- 30-40%	(3)
- 40-50%	(4)
- 50-60%	(5)
- 60-70%	(6)
- 80-90%	(7)
- 90-100% (8)
- Could not estimate (9)

Q3.2 What is the approximate, highest percent of active users during the implementation period?

- Less than 20%	(1)
- 20-30%	(2)
- 30-40%	(3)
- 40-50%	(4)
- 50-60%	(5)
- 60-70%	(6)
- 80-90%	(7)
- 90-100%	(8)
- Could not estimate (9)

Q108 Approximately, how many activations were made?

- Less than 100,000	(1)
- 100,000-200,000	(4)
- 200,000-300,000 (5)
- 300,000-400,000 (6)
- 400,000-500,000 (7)
- 500,000-1 million	(8)
- 1 million – 2 million (9)
- More than 2 million (10)
- Could not estimate (11)

Q3.3 Approximately, how many exposure notifications were made?

- Less than 100,000	(1)
- 100,000-200,000	(2)
- 200,000-300,000	(3)
- 300,000-400,000	(4)
- 400,000-500,000	(5)
- 500,000 - 1 million (6)
- More than million (8)
- Could not estimate (7)

Q109 How was the data above used in the pandemic response?

Q3.4 Do you think your EN program achieved your goals and objectives? Please describe in what ways

Q3.5 What are the “wishlist” resources that would have led to better programatic outcomes?___________

Q3.6 What are the “wishlist” resources that would have led to better health outcomes?____________

T3.3 **Identifying Barriers**

Q3.7 Please indicate your level of agreement with the following statements as barriers to your EN program.

|  | Strongly disagree (1) | Disagree (2) | Neutral (3) | Agree (4) | Strongly agree (5) | Not applicable (6) |
| --- | --- | --- | --- | --- | --- | --- |
| Lack of political support from policy makers (1) |  |  |  |  |  |  |
| Lack of departmental technical knowledge (2) |  |  |  |  |  |  |
| Lack of informatics infrastructure capacities within your PHA (3) |  |  |  |  |  |  |
| Lack of staff/workforce within your PHA (4) |  |  |  |  |  |  |
| Lack of IT support within your PHA (10) |  |  |  |  |  |  |
| Lack of organizational support from your health department (5) |  |  |  |  |  |  |
| Financial barriers (6) |  |  |  |  |  |  |
| Community mistrust in government (7) |  |  |  |  |  |  |
| Community mistrust in technology companies (8) |  |  |  |  |  |  |
| Community mistrust in EN system (9) |  |  |  |  |  |  |
| Difficulty in navigating legal systems related to EN technology development and implementation (11) |  |  |  |  |  |  |

Q3.8 If there is a barrier you would like to elaborate on, please describe below____________________

Q3.9 Are there other issues you would like to add?_____________________

Q3.10 What ramifications did you observe when the EN systems were turned off? (select all that apply)

- Difficulty to ramp up the program again (1)
- Loss of ability to collect data (4)
- Loss of surveillance metrics (5)
- Other (please describe) (6) __________________________________________________

End of Block: Section 3: Identifying Successes, Barriers, and Facilitators of EN Systems

Start of Block: Section 4a: Functionalities, Characteristics, and Design Considerations

T4a.1 **Section 4a: Functionalities, Characteristics, and Design Considerations**

Q4a.1 Of the following, rate your level of agreement with how important it is to incorporate each user functionality in the next version of EN application.

|  | Not very important (1) | Somewhat important (2) | Important (3) | Very important (4) |
| --- | --- | --- | --- | --- |
| Ability to self-report at-home tests (1) |  |  |  |  |
| Ability to turn off app without turning off Bluetooth (2) |  |  |  |  |
| Ability for users to receive messages when there is not a high-risk for exposure (3) |  |  |  |  |
| Ability to get additional public health information (I.e. testing and social services info) (4) |  |  |  |  |
| Bi-directional messaging (to and from public) (5) |  |  |  |  |
| Users set own personal risk score for when they want to be notified (6) |  |  |  |  |
| Ability to incorporate other personal risk factors (i.e. cancer, chronic lung disease, obesity, etc.) (7) |  |  |  |  |
| Non-individualized notification of disease outbreak (I.e. outbreak of E. coli in your area) (8) |  |  |  |  |
| Ability to efficiently customize risk score for different diseases and/or variants of the same pathogen (9) |  |  |  |  |

Q4a.2 Of the functionalities noted above, which do you think are the three most important for the next version of EN application? (Rank Order)

______ Ability to self-report at-home tests (1)

______ Ability to turn off app without turning off Bluetooth (2)

______ Ability for users to receive messages when there is not a high-risk for exposure (3)

______ Ability to get additional public health information (I.e. testing and social services info) (4)

______ Bi-directional messaging (to and from public) (5)

______ Users set own personal risk score for when they want to be notified (6)

______ Ability to incorporate other personal risk factors (7)

______ Non-individualized notification of disease outbreak (I.e. outbreak of E. coli in your area) (8)

Q4a.3 Are there additional functions not mentioned above that should be incorporated into the next version of EN?_________________________________________

Q4a.4 Of the following system characteristics, rate your level of agreement with how important it is to incorporate each to the next version of EN application.

|  | Not very important (1) | Somewhat important (2) | Important (3) | Very important (4) |
| --- | --- | --- | --- | --- |
| Centralized design (1) |  |  |  |  |
| Decentralized design (2) |  |  |  |  |
| Use of metadata to improve risk estimation (3) |  |  |  |  |
| Differential messaging based on level of risk (4) |  |  |  |  |
| Use of sensors (GPS, camera, microphone) to assess level of risk (5) |  |  |  |  |
| General venue-based notifications (I.e. concerts and sports events) (6) |  |  |  |  |
| Integration with other surveillance data streams to augment analytics and forecasting (7) |  |  |  |  |
| (8) |  |  |  |  |

Q4a.5 Of the key system characteristics noted above, which do you think are the three most important for the next version of EN? (Rank Order)

______ Centralized design (1)

______ Decentralized design (2)

______ Use of metadata to improve risk estimation (3)

______ Differential messaging based on level of risk (4)

______ Use of sensors (GPS, camera, microphone) to asses level of risk (5)

______ General venue-based notifications (I.e. concerts and sports events) (6)

______ Integration with other surveillance data streams to augment analytics and forecasting (7)

Q4a.6 Are there additional system characteristics not mentioned above that should be incorporated into the next version of EN application?___________________________________

Q4a.7 Which of the following do you believe is the best EN signaling system:

- Bluetooth Low Energy	(1)
- GPS-based	(2)
- Ultra-Wide band	(3)
- Ultrasound	(4)
- Mix (please describe) (5) __________________________________________________

Q4a.8 Why?____________________________________________

Q4a.9 Do you think custom hardware should be developed to improve EN? (i.e. Singapore developed and distributed devices to promote equity and improve distance readings)

- Yes (1)
- No (2)

Q4a.10 Why?_______________________________________________________________

Q4a.11 Are there ways to improve the opt-in sharing of information? Should there be different levels of sharing?____________________________

Q4a.12 How can we better inform PHAs about user experiences, behaviors, and biases while taking user privacy into consideration?_______________________

End of Block: Section 4a: Functionalities, Characteristics, and Design Considerations

Start of Block: Section 4b: Characteristics of Disease

T4b.1 **Section 4b: Characteristics of Disease**

Q4b.1 Which diseases should be included as part of the next version of EN (assuming it will use a similar technology and proximity-based approached used for COVID-19)? (Select all that apply)

- Continue with COVID-19	(1)
- Influenza	(2)
- Measles	(3)
- TB	(4)
- Strep throat	(5)
- Common Cold (6)
- Traveler's health (7)

Q4b.2 Are there additional diseases that should be included in the next version of EN that are not mentioned above?______________________________________

Q4b.3 What modes of transmission are most applicable to the existing proximity-based EN application?

|  | Not very applicable (1) | Somewhat applicable (2) | Applicable (3) | Very applicable (4) |
| --- | --- | --- | --- | --- |
| Airborne (1) |  |  |  |  |
| Fomite (2) |  |  |  |  |
| Bloodborne transmission (3) |  |  |  |  |
| STD (MPX, etc.) (4) |  |  |  |  |

Q4b.4 Are there additional modes of transmission that are applicable to the next version of EN that are not mentioned above?_________________________

Q4b.5 How important is it to include the following Epi/clinical considerations when determining which pathogens to include for the next version of EN?

|  | Not very important (1) | Somewhat important (2) | Important (3) | Very important (4) |
| --- | --- | --- | --- | --- |
| Disease burden/prevalence (1) |  |  |  |  |
| Level of community immunity (2) |  |  |  |  |
| Community available to take protective actions (3) |  |  |  |  |
| Economic availability to take protective actions (4) |  |  |  |  |
| Transmissibility of the pathogen (i.e. incubation period, infectiousness, etc.) (5) |  |  |  |  |

Q4b.6 Are there additional epidemiological/clinical considerations that are not mentioned above? ____

End of Block: Section 4b: Characteristics of Disease

Start of Block: Section 4c: Macro-level Considerations

T4c.1 **Section 4c: Macro-level Considerations in Policy, Political Governance, and Global Partnerships**

Q4c.1 Where should the EN program be placed within an organizational/departmental structure?

- CI/CT workflow/program	(1)
- Data systems/informatics	(2)
- Emergency response	(3)
- Hybrid model (4)

Q4c.2 Are there additional structures where an EN program could be included?____________________

Q4c.3 Do you have suggestions as to what governance principles and procedures should be in place prior to the next implementation of the EN system?________________________________

Q4c.4 In the United States, do you think EN should be implemented nationally, on a state-by-state basis, or a hybrid of the two?

- Nationally	(1)
- State-by-state	(2)
- National as a default with an option for states to customize (3)
- Locally (4)
- Other (please describe) (5) __________________________________________________

Q4c.5 Why?________________________________________________________________

Q4c.6 How can the different states/agencies adopting EN systems organize the working groups, like the Digital Notification Alliance (DNA), better?_______________________

Q4c.7 How can EN implementors better utilize working groups?_____________________________

Q4c.8 What other national institutions should EN implementors collaborate with? Select all that apply.

- World Health Organization	(1)
- FEMA	(2)
- National Institute for Health	(3)
- Other (please describe) (4) __________________________________________________

Q4c.9 What other institutions can EN implementors look to for guidance on how to organize major groups, propose national policy, create technical documentation, etc.?_______________________

Q4c.10 How can those with experience implementing EN systems help other municipalities start similar systems within their jurisdictions?____________________________

Q4c.11 Should it be a high-priority to work directly with smartphone manufactures to expand current EN technology?

- Yes (1)
- No (2)

Q4c.12 Why?_______________________________________________________________

Q4c.13 If historic competitors, like Apple and Google, decide to no longer collaborate on EN technology, what other companies or types of technology can be utilized to allow PHAs to adopt EN systems?

End of Block: Section 4c: Macro-level Considerations

Start of Block: Section 4d: Privacy, Equity and Health Education Considerations

T4d.1 **Section 4d: Privacy, Equity and Health Education Considerations**

Q4d.1 During your experience in the implementation of EN within your jurisdiction, how did adoption change as COVID-19 language and guidance changed throughout the pandemic?__________

Q4d.2 Public trust, or lack thereof, had an essential influence on adoption levels of EN systems within respective jurisdictions. How would you prioritize establishing trust in the following institutions in order to increase adoption levels moving forward? (Rank order)

______ State government (1)

______ Federal government (2)

______ Technology companies (3)

______ EN system/technology (4)

______ Public Health Departments (5)

Q4d.3 In the following section, identify the option within each row that is more important to consider in relation to the level of privacy.

|  | Privacy is more important (1) | Privacy is less important (2) |
| --- | --- | --- |
| Accuracy of EN (1) |  |  |
| Perceived risk by users and their willingness to adopt (2) |  |  |
| Scalability tradeoff (3) |  |  |
| System evaluation capabilities (4) |  |  |

Q4d.4 What methods can PHAs use to improve trust in order to increase adoption?_____________

Q4d.5 When developing communications concerning a user’s exposure, which do you think is more important: accuracy of information or readability?

- Medical accuracy (1)
- Readability (2)

Q4d.5 Why?________________________________________________________________

Q4d.6 How important is it to prioritize public-facing communication campaigns concerning the following topic?

|  | Not very important (1) | Somewhat important (2) | Important (3) | Very important (4) |
| --- | --- | --- | --- | --- |
| How does EN technology work (1) |  |  |  |  |
| How to use the system (i.e. how to add your phone, how to report, etc.) (2) |  |  |  |  |
| What can individuals gain from using the system (3) |  |  |  |  |
| What do communities gain from utilizing the system (4) |  |  |  |  |
| When a person will and will not receive an EN (when are you considered exposed via the risk model) (5) |  |  |  |  |
| How user privacy is protected (6) |  |  |  |  |
| Reminders to use the system when testing positive (7) |  |  |  |  |
| Interoperability between states/countries (8) |  |  |  |  |

Q4d.7 Of the campaign topics listed above, which do you think are the three most important when implementing the next version of EN? (Rank order)

______ How EN technology works (1)

______ How to use the system (2)

______ What can individuals gain from using the system (3)

______ What can communities gain from utilizing the system (4)

______ When a person will and will not receive an EN (5)

______ How user privacy is protected (6)

______ Reminders to use the system when testing positive (7)

______ Interoperability between states/countries (8)

Q4d.8 Are there other messaging campaigns to include in future marketing strategies in order to help increase adoption and use?___________________________________________________

Q4d.9 How can PHAs best market EN technology if the app is reestablished during the next emergency?

Q4d.10 How can PHAs best market EN technology in times of normalcy if the app is reestablished prior to the next emergency?_____________________________________________________________

Q4d.11 How can system implementors account for constituents that are less digitally savvy or don’t have access to smartphone devices?________________________________________________

Q4d.12 How did your program interact with your users in an accessible way? (Select all that apply)

- Public health call center/help desk	(1)
- Online chat-box	(2)
- CI/CT caller integration	(3)
- Direct email availability	(4)
- Other (please describe) (5) __________________________________________________

Q4d.13 Rank which strategies would best ease user burden.

______ Different user workflows (1)

______ Easier methods for activation (2)

______ More accessible language (3)

______ Increased access to other public health resources (4)

______ New EN hardware (5)

______ New methods for direct user communication (I.e. help desk) (6)

Q4d.14 Are there other opportunities to ease user burden?___________________________________

End of Block: Section 4d: Privacy, Equity and Health Education Considerations

Start of Block: Section 5: Broadening the Scope of the Smartphone Application

T5.1 **Section 5: Broadening the Scope of the Smartphone Application**

T5.2 *There is potential to broaden the scope of the smartphone application to include early warning systems for public health disasters (I.e. wildfires, mass shootings, earthquakes, floods, etc.) and individual-level preventive measures (I.e. fall notifications, etc.).*

Q5.1 Do you think it makes sense to make a more comprehensive EN app?

- Yes	(1)
- No	(2)
- Other (please describe) (3) __________________________________________________

Q5.2 If you think it is a possibility to create a comprehensive EN app, what level of priority would it be?

- Immediate priority	(1)
- Mid-term priority	(2)
- Long-term priority (3)

Q5.3 What are the potential barriers to broadening the scope of this application?__________________

End of Block: Section 5: Broadening the Scope of the Smartphone Application

Start of Block: Section 6: SME Referrals and Participant Demographics

T6.1 **Section 6: SME Referrals and Participant Demographics**

Q6.1 Do you know someone who would be interested in and good fit for participating in this survey?

- Yes (please share their email below) (1) _____________________________
- No (2)
- Maybe; requires additional information (please share their email below) (3) _________

Q6.2 Did you work on this survey with your team in your jurisdiction?

- Yes (1)
- No (2)

Q6.3 What is your age range?

- 18-20 years (1)
- 21-29 years (2)
- 30-39 years (3)
- 40-49 years (4)
- 50-59 years (5)
- 60 years or older (6)

Q6.4 What is your World Health Organization (WHO) region?

- African Region (AFR)	(1)
- Region of the Americas (AMR)	(2)
- South-East Asian Region (SEAR)	(3)
- European Region (EUR)	(4)
- Eastern Mediterranean Region (EMR)	(5)
- Western Pacific Region (WPR) (6)

Q6.5 Optional: If you are US based, which CDC region are you based in?

- Region 1: Connecticut, Maine, Massachusetts, New Hampshire, Rhode Island, and Vermont (1)
- Region 2: New Jersey, New York, The Commonwealth of Puerto Rico, and The U.S. Virgin Islands (4)
- Region 3: Delaware, District of Columbia, Maryland, Pennsylvania, Virginia, and West Virginia (5)
- Region 4: Alabama, Florida, Georgia, Kentucky, Mississippi, North Carolina, South Carolina, and Tennessee (6)
- Region 5: Illinois, Indiana, Michigan, Minnesota, Ohio, and Wisconsin (7)
- Region 6: Arkansas, Louisiana, New Mexico, Oklahoma, and Texas (8)
- Region 7: Iowa, Kansas, Missouri, and Nebraska (9)
- Region 8: Colorado, Montana, North Dakota, South Dakota, Utah, and Wyoming (10)
- Region 9: Arizona, California, Hawaii, Nevada, Commonwealth of the Northern Mariana Islands (CNMI), U.S. Territory of Guam, Marshall Islands, Palau, American Samoa. (11)
- Region 10: Alaska, Idaho, Oregon, and Washington and Tribal Nations (12)

Q6.6 Optional: What country and/or state did you work for in pandemic response?_________________

End of Block: Section 6: SME Referrals and Participant Demographics
